# Chronic Suppurative Pyelitis

**Published:** 1889-12-28

**Authors:** 


### CHRONIC SUPPURATIVE PYELITIS.

Mr. Reginald Harrison, surgeon to St. Peter's Hospital, contributes to the *Lancet*, December 7, a paper on the treatment of some forms of chronic suppurating kidneys by perinœal puncture and drainage. Instances occur when backward pressure of urine leads not only to dilatation of the ureters and kidneys, but to extensive suppuration. In some of these conditions the reduction of the obstruction in the urethra by systematic dilatation is followed by improvement. In other cases improvement does not follow. A case of the kind determined Mr. Harrison to drain the suppurating parts by puncturing the perinœum, and putting a large drainage tube into the bladder. Mr. Harrison has now operated in about ten cases of chronic suppurative pyelitis, involving both kidneys. And Mr. Harrison summarises as follows: That in a large number of cases of simple suppurative pyelitis caused by obstruction below, the parts recover as the resistance to the urine is reduced. That advanced forms of chronic double suppurative pyelitis are best treated by an opening in the perinœum.

